# Supplementary figures and images for: Forward optic flow is prioritised in visual awareness independently of walking direction
Source: PLoS One. 2021 May 4;16(5):e0250905. doi: 10.1371/journal.pone.0250905 (PMC8096117; doi:10.1371/journal.pone.0250905)

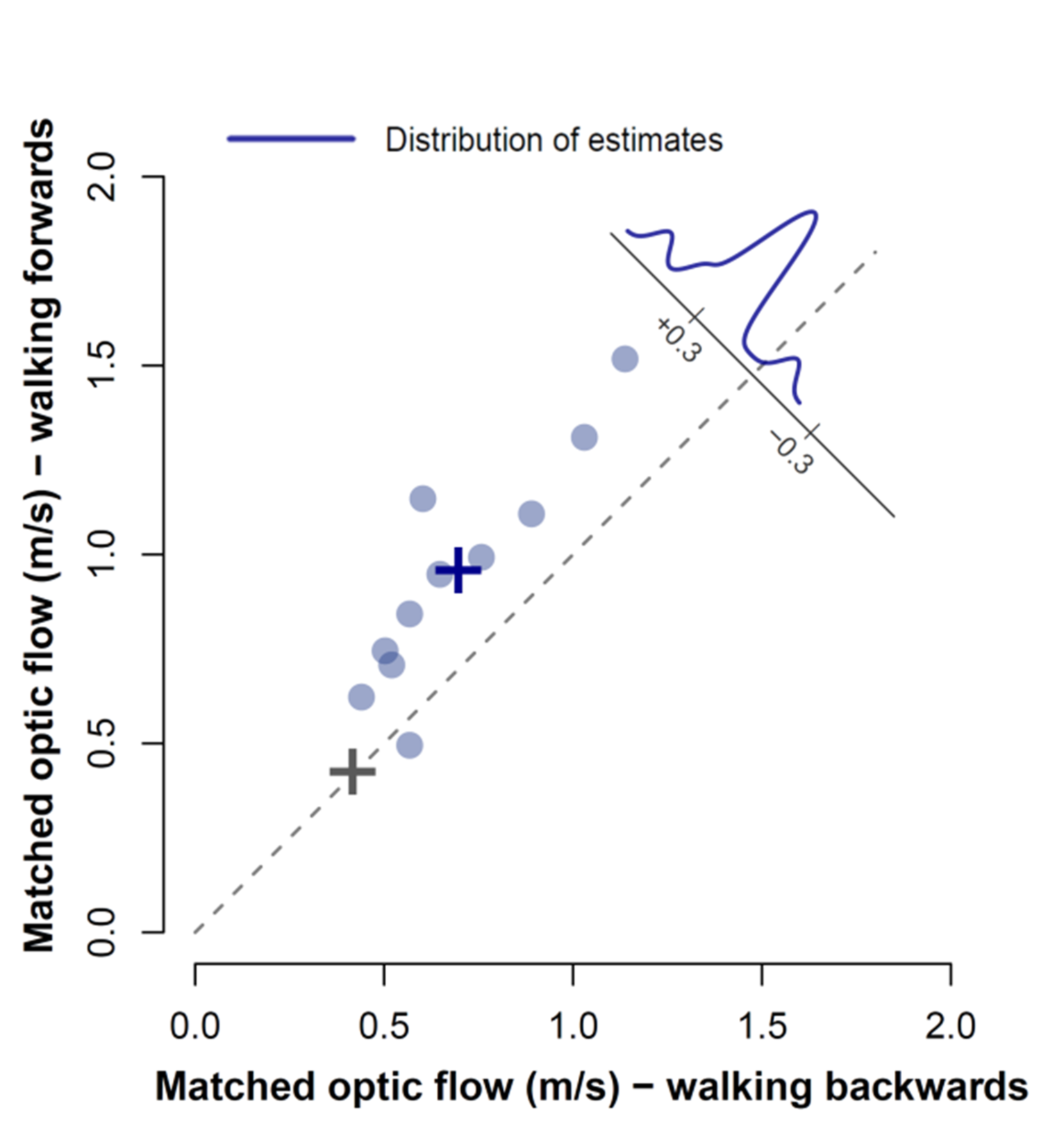

Supplement: S1 Fig — Each dot’s coordinates represent a participant’s mean matched optic flow speed when walking backwards (x-axis) and walking forwards (y-axis). Coordinates of the blue cross represent the sample means from both conditions. The gray cross indicates the physically accurate optic flow speed for the walking pace used. The dashed line represents optic flow speeds being equal on both walking conditions. The results indicate an overall overestimation of matched optic flow speed (as compared to the locomotion velocity) in both conditions; however, this tendency is more pronounced in the forward-walking condition–the probability distribution of estimates shifts toward higher values for walking forwards than for walking backwards. (TIFF) [file pone.0250905.s001.tiff]

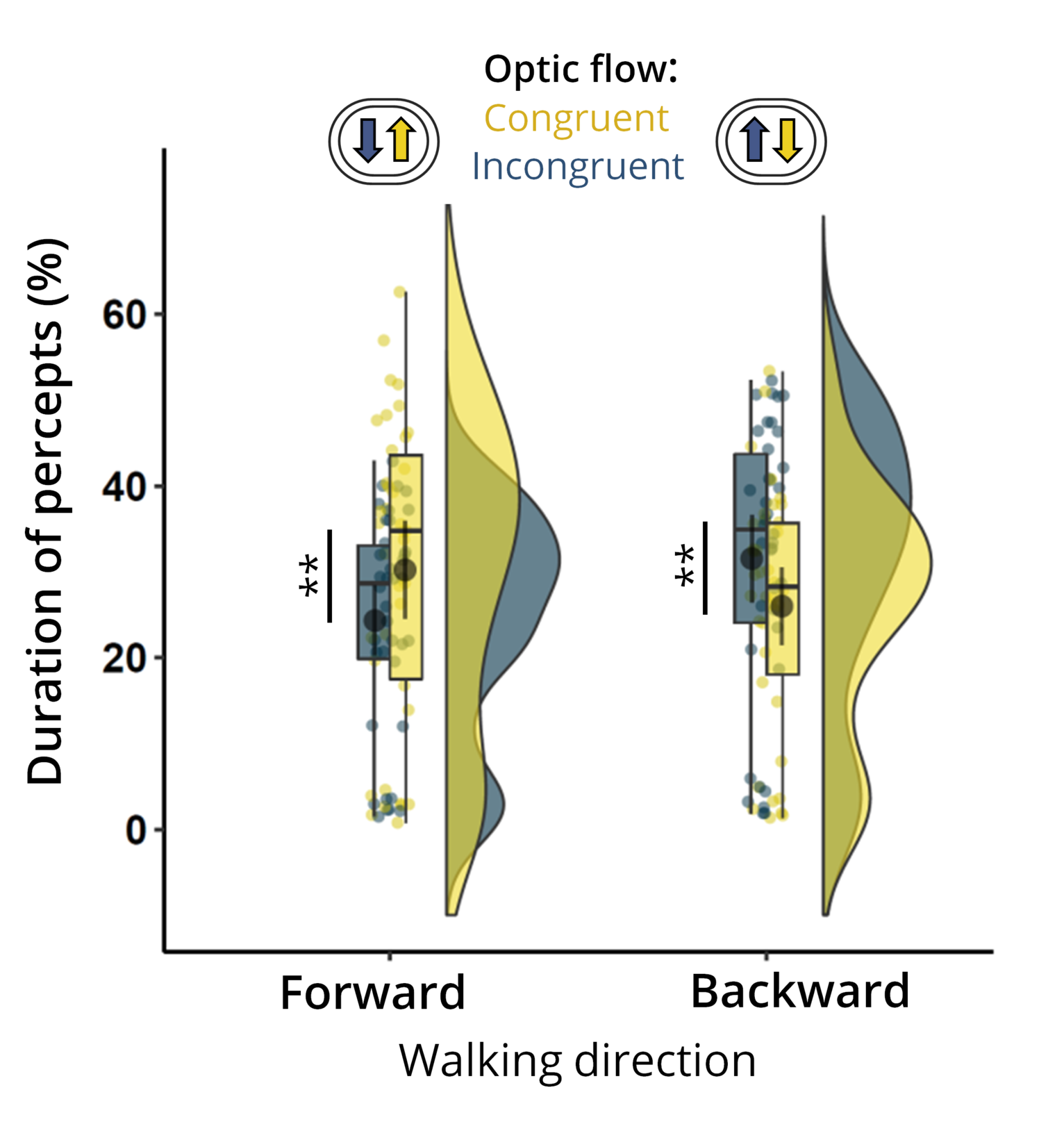

Supplement: S2 Fig — Expanding optic flow (congruent with forward locomotion and incongruent with backward movement) predominated visual awareness independently of walking direction. **p < 0.01. (TIFF) [file pone.0250905.s002.tiff]

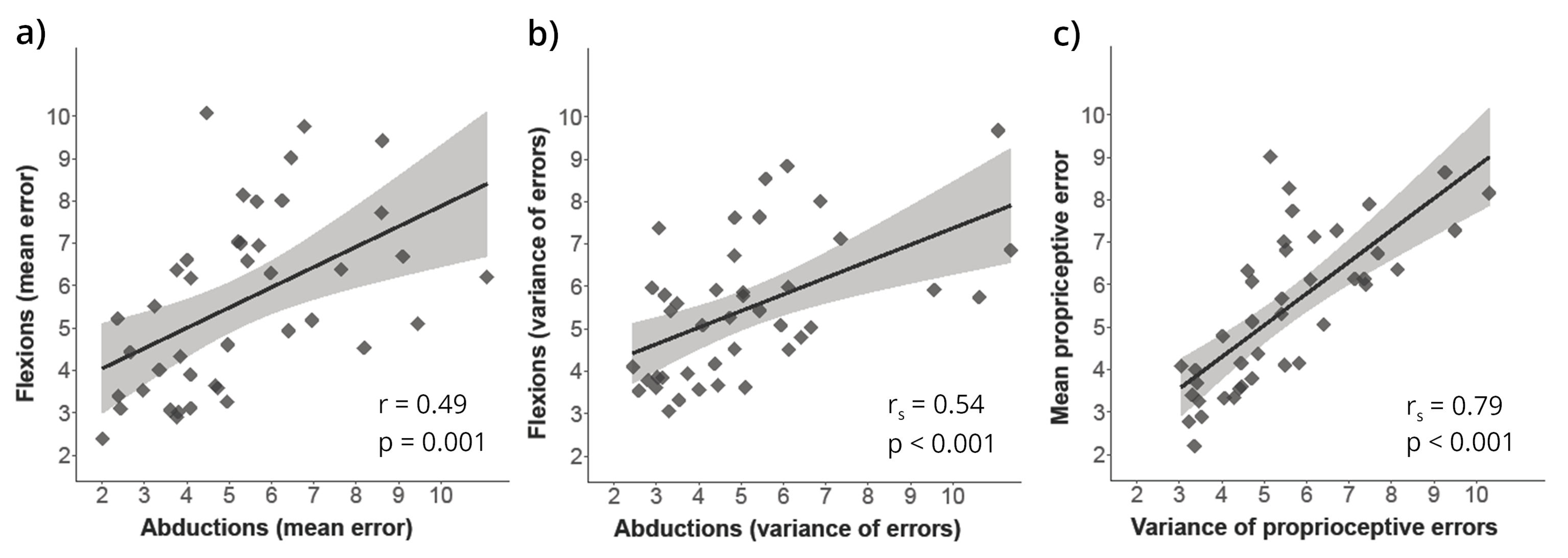

Supplement: S3 Fig — The positive correlations between performances for flexions and abductions in terms of (a) proprioceptive accuracy (mean proprioceptive error) and (b) proprioceptive precision (variance of proprioceptive errors). (c) A strong overall correlation between proprioceptive accuracy and proprioceptive precision. (TIFF) [file pone.0250905.s003.tiff]

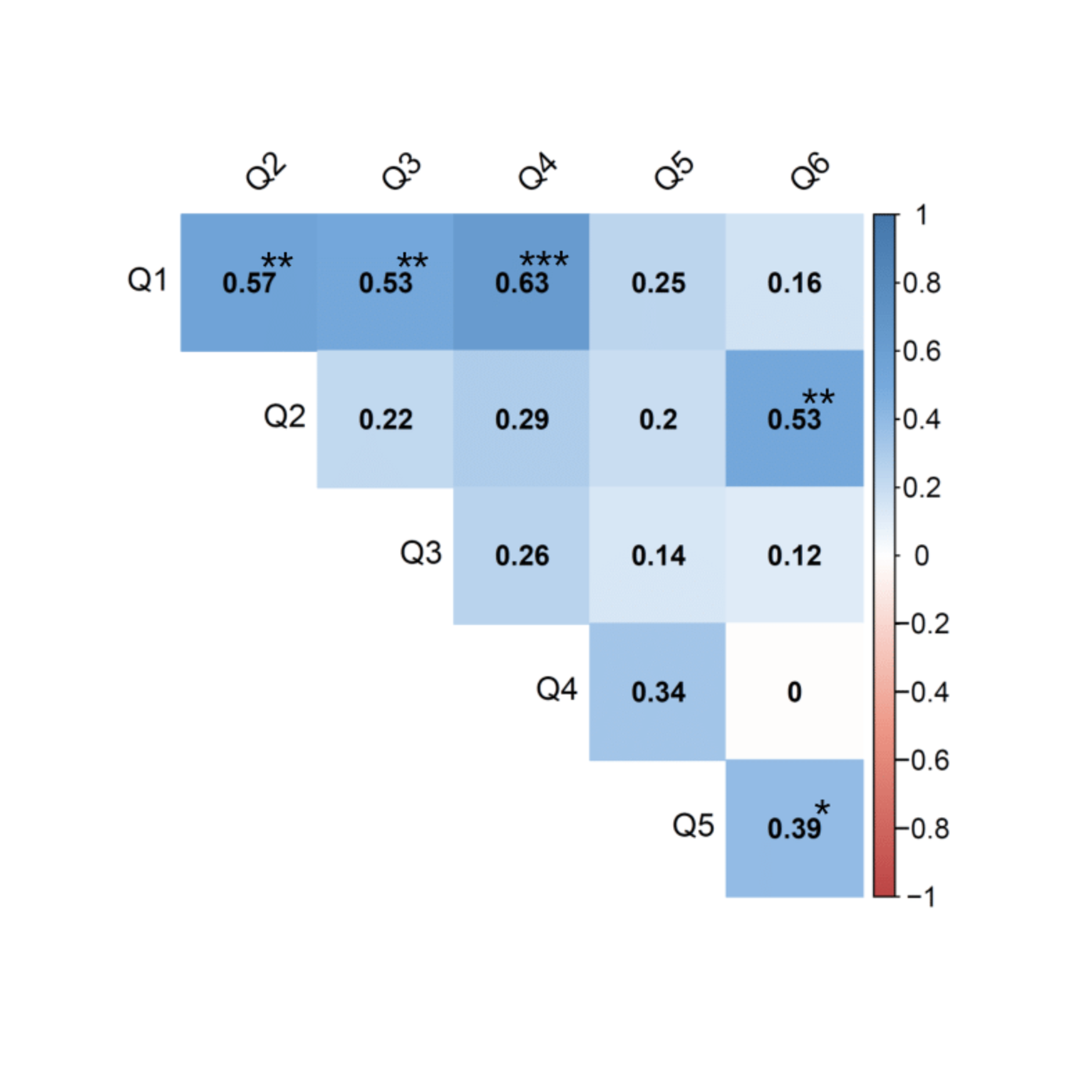

Supplement: S4 Fig — *** p < 0.001; ** p < 0.01; * p < 0.05. (TIFF) [file pone.0250905.s004.tiff]

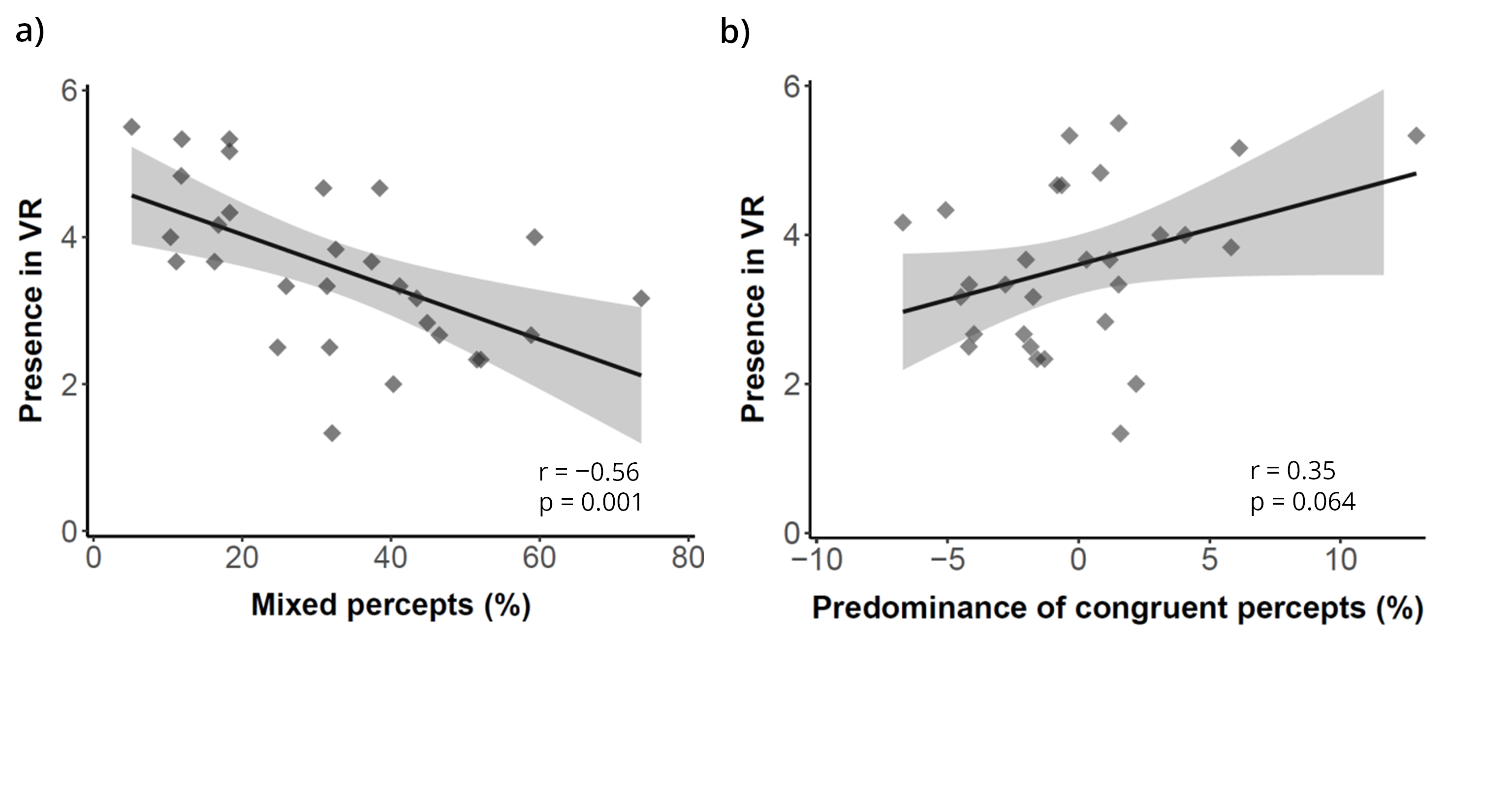

Supplement: S5 Fig — (a) Sense of presence was negatively correlated with the proportion of mixed percepts, and (b) showed a tendency to be positively correlated with the degree of predominance of locomotion-congruent optic flows. (TIFF) [file pone.0250905.s005.tiff]

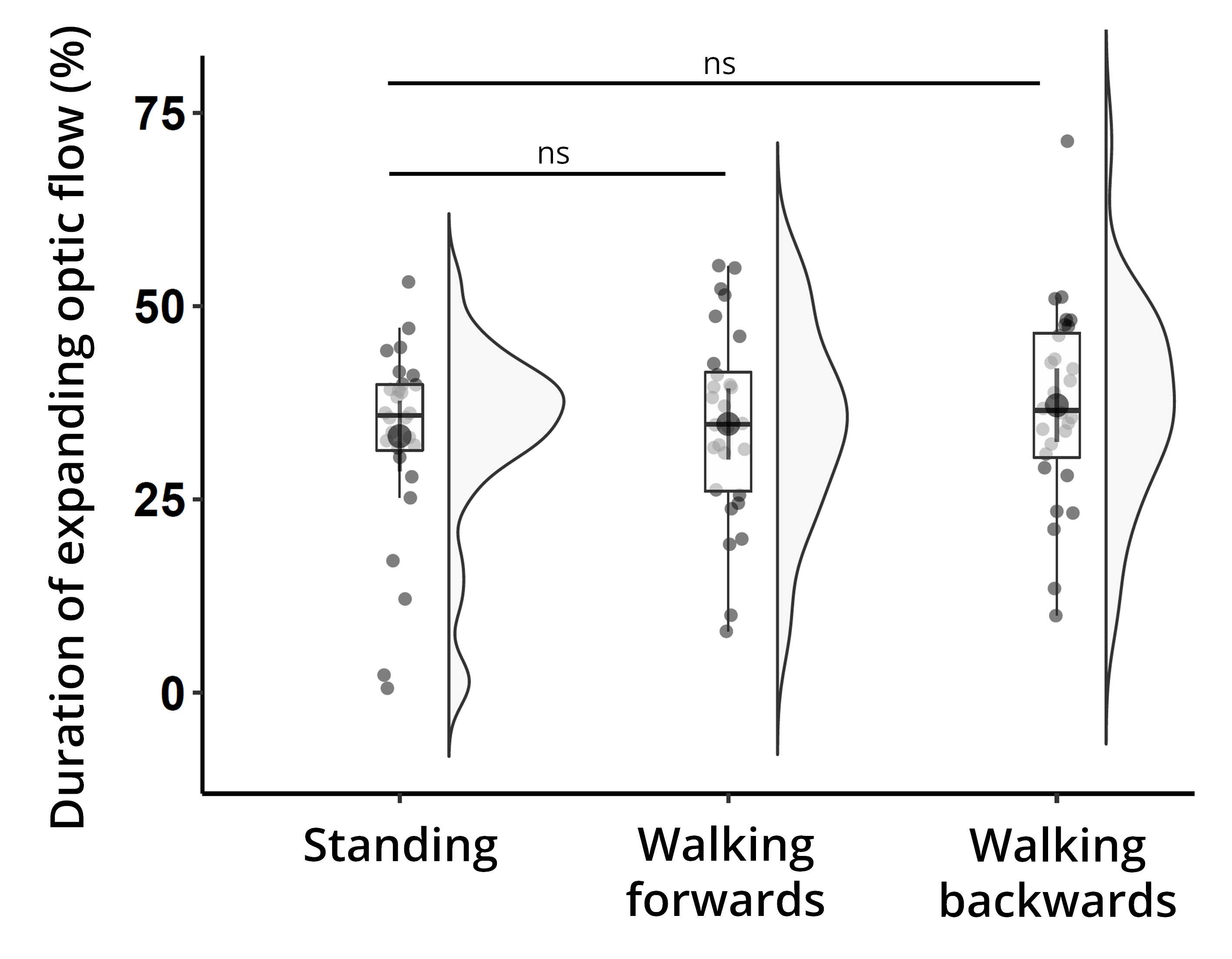

Supplement: S6 Fig — No significant differences were found between training (standing) blocks and visually identical blocks with either forward or backward self-motion. (TIF) [file pone.0250905.s006.tif]

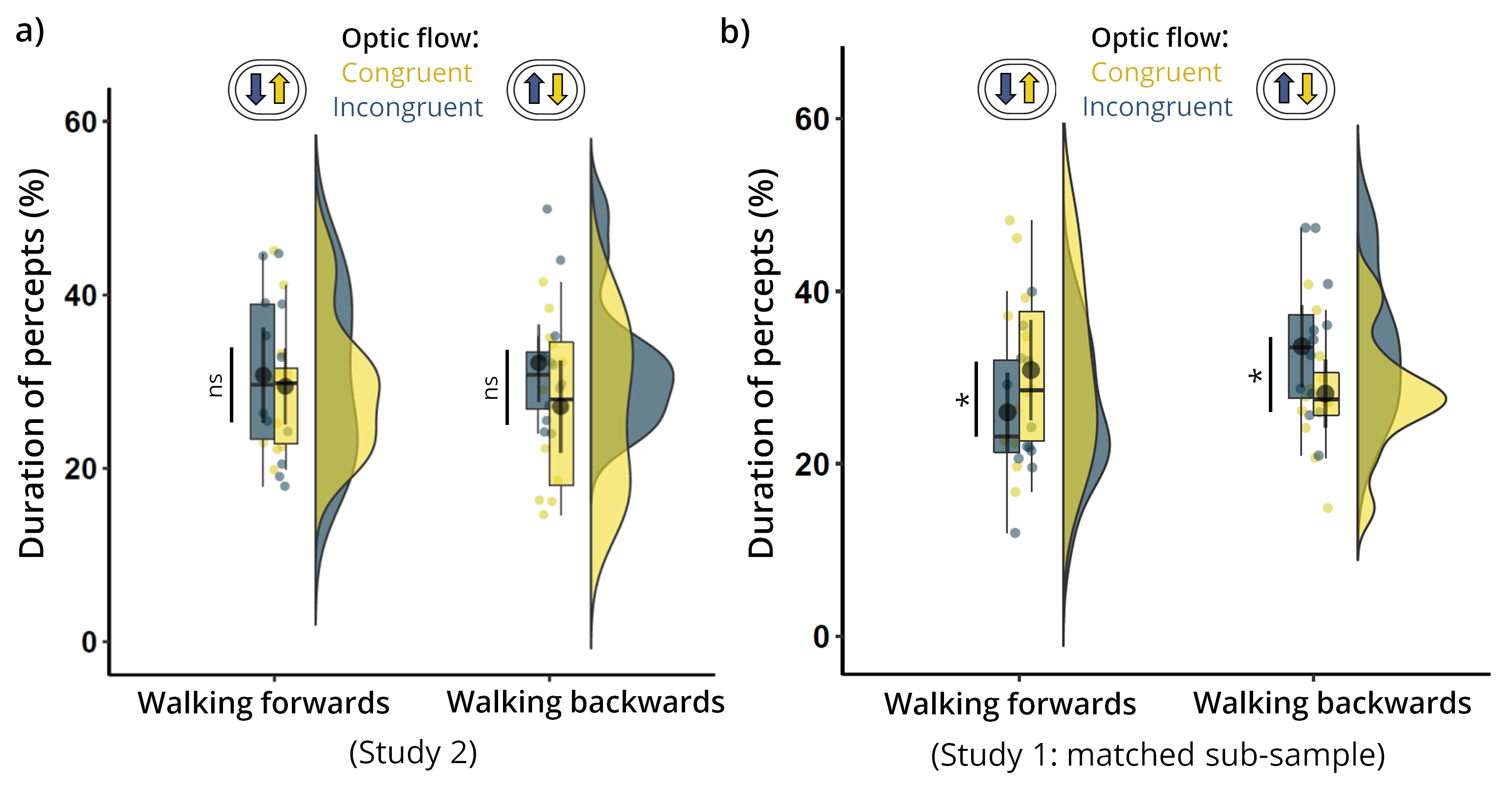

Supplement: S7 Fig — (a) Study 2: when contracting optic flow (congruent with backward locomotion) was faster than expanding flow (congruent with forward movement), no significant differences in cumulative durations of percepts were found in either walking condition (p values > 0.283). (b) Study 1 (sub-sample matched with respect to the distribution of mixed percepts in study 2): when expanding optic flow was faster than contracting flow, it dominated visual awareness independently of walking direction (p values < 0.022). * p < 0.05; ns = non-significant. (TIFF) [file pone.0250905.s007.tiff]
