# Supplementary material for: Forward optic flow is prioritised in visual awareness independently of walking direction
Source: PLoS One. 2021 May 4;16(5):e0250905. doi: 10.1371/journal.pone.0250905 (PMC8096117; doi:10.1371/journal.pone.0250905)
Supplement: S1 Table — The subject is instructed: “Please rate the extent to which you are experiencing now each of the symptoms”. (DOCX) [file pone.0250905.s008.docx]

| **Symptom** | **Severity** | | | |
| --- | --- | --- | --- | --- |
| General discomfort | *None* | *Slight* | *Moderate* | *Severe* |
| Headache | *None* | *Slight* | *Moderate* | *Severe* |
| Nausea | *None* | *Slight* | *Moderate* | *Severe* |
| Difficulty concentrating | *None* | *Slight* | *Moderate* | *Severe* |
| Blurred vision | *None* | *Slight* | *Moderate* | *Severe* |
| Dizziness | *None* | *Slight* | *Moderate* | *Severe* |
| Confusion | *None* | *Slight* | *Moderate* | *Severe* |
