# Supplementary material for: Forward optic flow is prioritised in visual awareness independently of walking direction
Source: PLoS One. 2021 May 4;16(5):e0250905. doi: 10.1371/journal.pone.0250905 (PMC8096117; doi:10.1371/journal.pone.0250905)
Supplement: S2 Table — (DOCX) [file pone.0250905.s009.docx]

| **Question** | **Scale description** |
| --- | --- |
| Q1. Please rate your sense of being in the virtual space (in the tunnel), on the following scale from 1 to 7, where 7 represents your normal experience of being in a place. I had a sense of “being there” in the virtual space | *1. Not at all  ... 7. Very much.* |
| Q2. To what extent were there times during the experience when the virtual space was the reality for you? There were times during the experience when the virtual space was the reality for me... | *1. At no time*  *...*  *7. Almost all the time.* |
| Q3. When you think back about your experience, do you think of the virtual space more as images that you saw, or more as somewhere that you visited? The virtual space seems to me to be more like… | *1. Images that I saw  ...  7. Somewhere that I visited.* |
| Q4. During the time of the experience, which was strongest on the whole, your sense of being in the virtual space, or of being elsewhere? I had a stronger sense of… | *1. Being elsewhere  ...  7. Being in the virtual space.* |
| Q5. Consider your memory of being in the virtual space. How similar in terms of the structure of the memory is this to the structure of the memory of other places you have been today? By ‘structure of the memory’ consider things like the extent to which you have a visual memory of the virtual space, whether that memory is in colour, the extent to which the memory seems vivid or realistic, its size, location in your imagination, the extent to which it is panoramic in your imagination, and other such structural elements. I think of the virtual space as a place in a way similar to other places that I've been today… | *1. Not at all  ...  7. Very much so.* |
| Q6. During the time of the experience, did you often think to yourself that you were actually in the tunnel? During the experience I often thought that I was really standing in the tunnel... | *1. Not very often  ...  7. Very much so.* |

.
