# Supplementary material for: Forward optic flow is prioritised in visual awareness independently of walking direction
Source: PLoS One. 2021 May 4;16(5):e0250905. doi: 10.1371/journal.pone.0250905 (PMC8096117; doi:10.1371/journal.pone.0250905)
Supplement: S1 File — (DOCX) [file pone.0250905.s010.docx]

**Supporting Information**

Forward optic flow is prioritised in visual awareness
 independently of walking direction

Paweł Motyka^1^*, Mert Akbal^2,3^, Piotr Litwin^1^

^1^Faculty of Psychology, University of Warsaw, Warsaw, Poland
^2^Department of Neurology, Max Planck Institute for Human Cognitive and Brain Sciences, Leipzig, Germany
^3^Academy of Fine Arts Saar, Saarbrücken, Germany

*Corresponding author:
E-mail: pawel.motyka@psych.uw.edu.pl

**Supplementary method**

**S1 Table. Shortened version of the Simulator Sickness Questionnaire (SSQ).** The subject is instructed: “Please rate the extent to which you are experiencing now each of the symptoms”.

| **Symptom** | **Severity** | | | |
| --- | --- | --- | --- | --- |
| General discomfort | *None* | *Slight* | *Moderate* | *Severe* |
| Headache | *None* | *Slight* | *Moderate* | *Severe* |
| Nausea | *None* | *Slight* | *Moderate* | *Severe* |
| Difficulty concentrating | *None* | *Slight* | *Moderate* | *Severe* |
| Blurred vision | *None* | *Slight* | *Moderate* | *Severe* |
| Dizziness | *None* | *Slight* | *Moderate* | *Severe* |
| Confusion | *None* | *Slight* | *Moderate* | *Severe* |

**S2 Table. Adapted version of the Slater-Usoh-Steed Questionnaire (SUS).**

| **Question** | **Scale description** |
| --- | --- |
| Q1. Please rate your sense of being in the virtual space (in the tunnel), on the following scale from 1 to 7, where 7 represents your normal experience of being in a place. I had a sense of “being there” in the virtual space | *1. Not at all  ... 7. Very much.* |
| Q2. To what extent were there times during the experience when the virtual space was the reality for you? There were times during the experience when the virtual space was the reality for me... | *1. At no time*  *...*  *7. Almost all the time.* |
| Q3. When you think back about your experience, do you think of the virtual space more as images that you saw, or more as somewhere that you visited? The virtual space seems to me to be more like… | *1. Images that I saw  ...  7. Somewhere that I visited.* |
| Q4. During the time of the experience, which was strongest on the whole, your sense of being in the virtual space, or of being elsewhere? I had a stronger sense of… | *1. Being elsewhere  ...  7. Being in the virtual space.* |
| Q5. Consider your memory of being in the virtual space. How similar in terms of the structure of the memory is this to the structure of the memory of other places you have been today? By ‘structure of the memory’ consider things like the extent to which you have a visual memory of the virtual space, whether that memory is in colour, the extent to which the memory seems vivid or realistic, its size, location in your imagination, the extent to which it is panoramic in your imagination, and other such structural elements. I think of the virtual space as a place in a way similar to other places that I've been today… | *1. Not at all  ...  7. Very much so.* |
| Q6. During the time of the experience, did you often think to yourself that you were actually in the tunnel? During the experience I often thought that I was really standing in the tunnel... | *1. Not very often  ...  7. Very much so.* |

.

**Supplementary results**

**
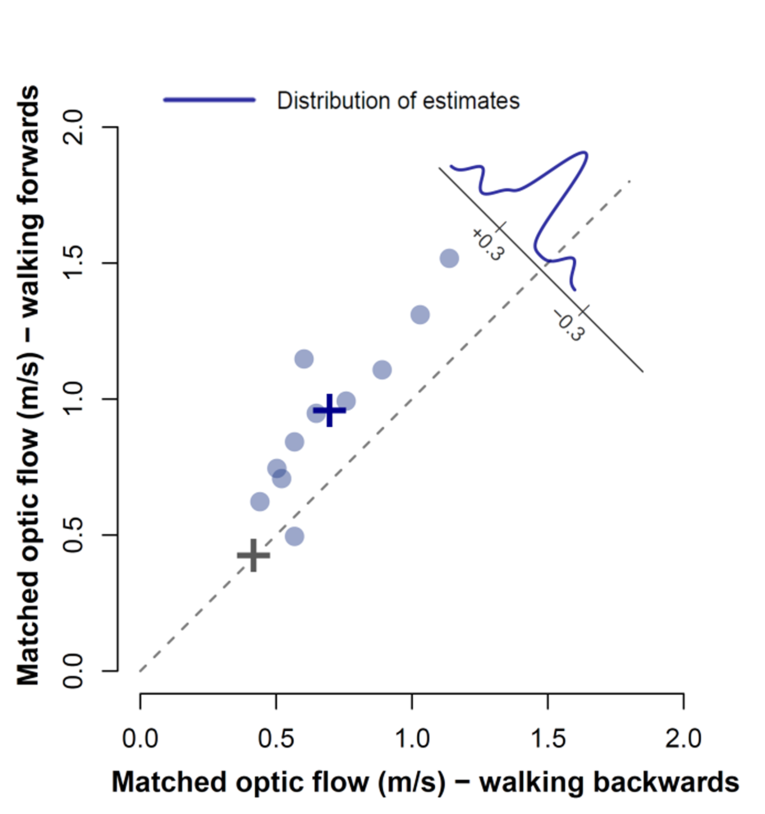
**

**S1 Fig. Optic flow speed values chosen as being subjectively matched to velocity of walking (0.42 m/s).** Each dot’s coordinates represent a participant’s mean matched optic flow speed when walking backwards (x-axis) and walking forwards (y-axis). Coordinates of the blue cross represent the sample means from both conditions. The gray cross indicates the physically accurate optic flow speed for the walking pace used. The dashed line represents optic flow speeds being equal on both walking conditions. The results indicate an overall overestimation of matched optic flow speed (as compared to the locomotion velocity) in both conditions; however, this tendency is more pronounced in the forward-walking condition – the probability distribution of estimates shifts toward higher values for walking forwards than for walking backwards.

**Effects of locomotion on perceptual awareness of optic flow patterns (unfiltered sample).** For the sample without any exclusions, there was no main effect of congruency (F(1, 37) = 0.04, p = 0.845, η^2^_G_ < 0.001), the main effect of walking reached the threshold of significance (F(1, 37) = 5.96, p = 0.020, η^2^_G_ = 0.002) and there was a significant interaction between both factors (F(1, 37) = 37.0, p < 0.001, η^2^_G_ = 0.030; Fig S1). Post hoc Bonferroni-corrected comparisons indicated a pattern of results equivalent to the one observed in the main analysis: for walking forwards, congruent optic flow was perceived longer (M = 30.2%, SD = 17.8%) than incongruent flow (M = 24.3%, SD = 12.7%, t(37) = 4.53, p = 0.001), whereas for walking backwards, incongruent optic flow (M = 31.5%, SD = 16.0%) predominated over congruent flow (M = 26.0%, SD = 14.2%, t(37) = 4.26, p = 0.004). The perceptual availability of expanding optic flow did not differ significantly between walking conditions (t(37) = 1.21, p = 1.000) and the same was found for contracting optic flow (t(37) = 1.54, p = 0.772). As in the main analysis, there was no significant association between the degree of predominance of congruent percepts and the variance of proprioceptive errors (r_s_(36) = 0.02, p = 0.882).

**
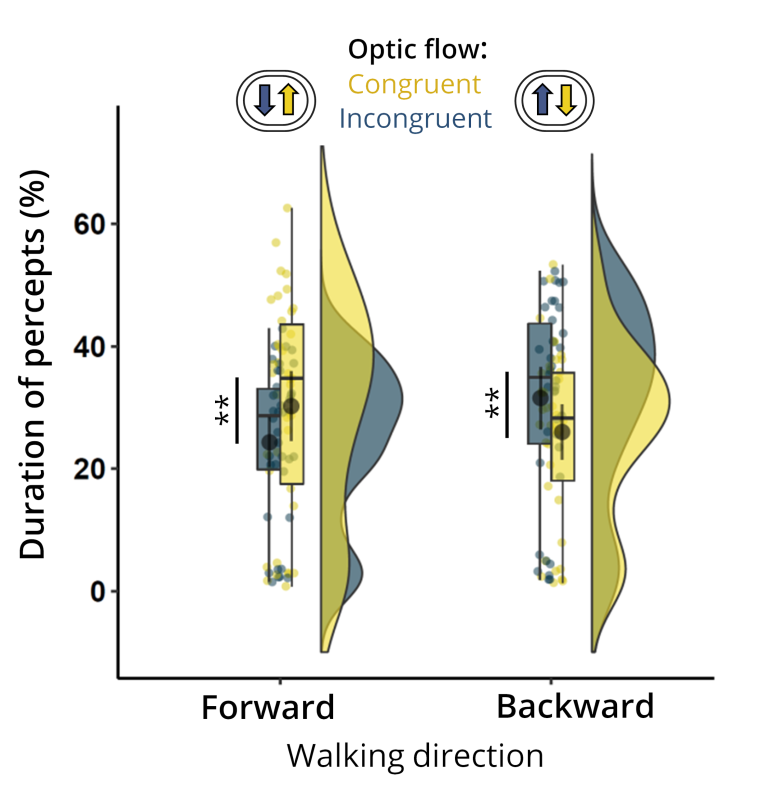
**

**S2 Fig. Locomotion effects on perceptual awareness for optic flow patterns (unfiltered sample**). Expanding optic flow (congruent with forward locomotion and incongruent with backward movement) predominated visual awareness independently of walking direction. **p < 0.01

**
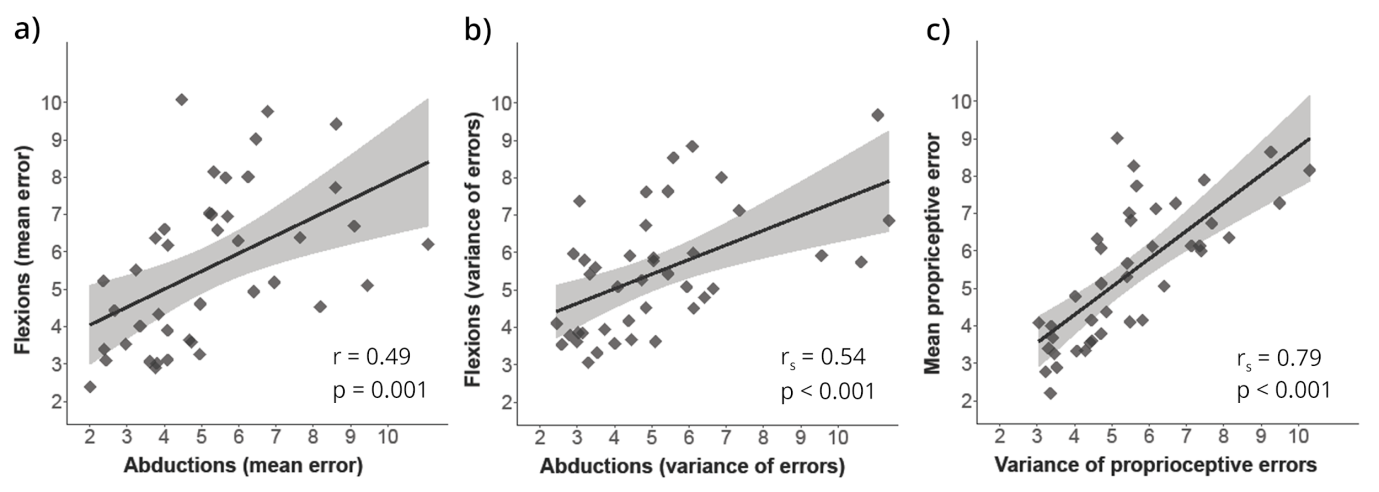
**

**S3 Fig. Proprioceptive assessment results.** The positive correlations between performances for flexions and abductions in terms of (**a**) proprioceptive accuracy (mean proprioceptive error) and (**b**) proprioceptive precision (variance of proprioceptive errors). (**c**) A strong overall correlation between proprioceptive accuracy and proprioceptive precision.

**
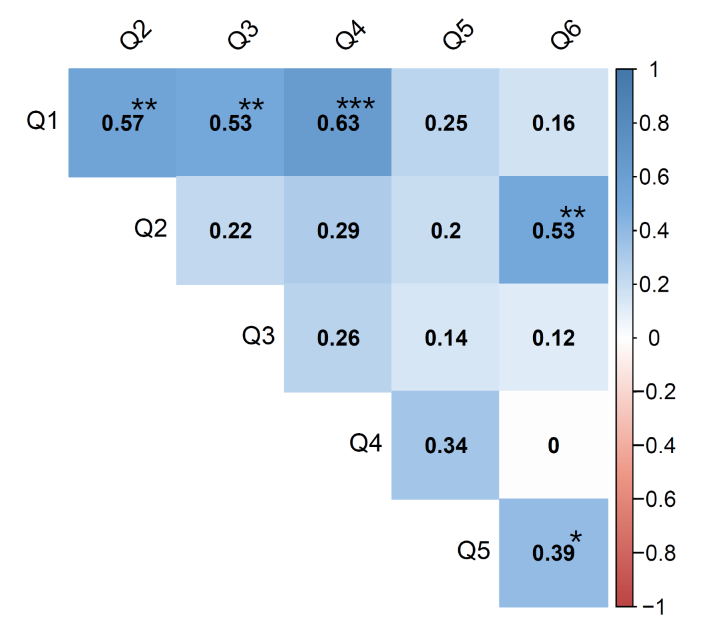


S4 Fig. Intercorrelations between items of the SUS questionnaire assessing sense of presence in the VR environment.** *** p < 0.001; ** p < 0.01; * p < 0.05

**
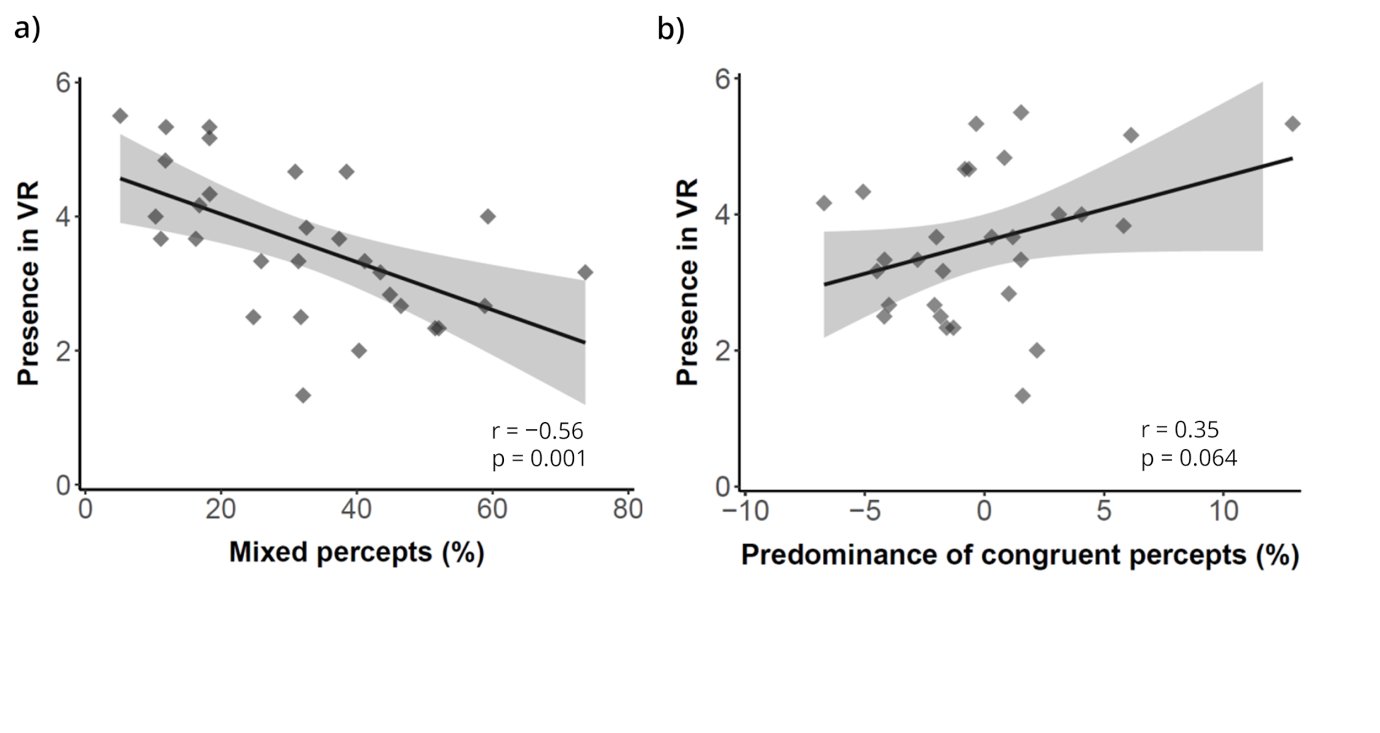
**

**S5 Fig. Associations between self-reported sense of presence in the virtual environment and the contents of visual awareness**. (**a**) Sense of presence was negatively correlated with the proportion of mixed percepts, and (**b**) showed a tendency to be positively correlated with the degree of predominance of locomotion-congruent optic flows.


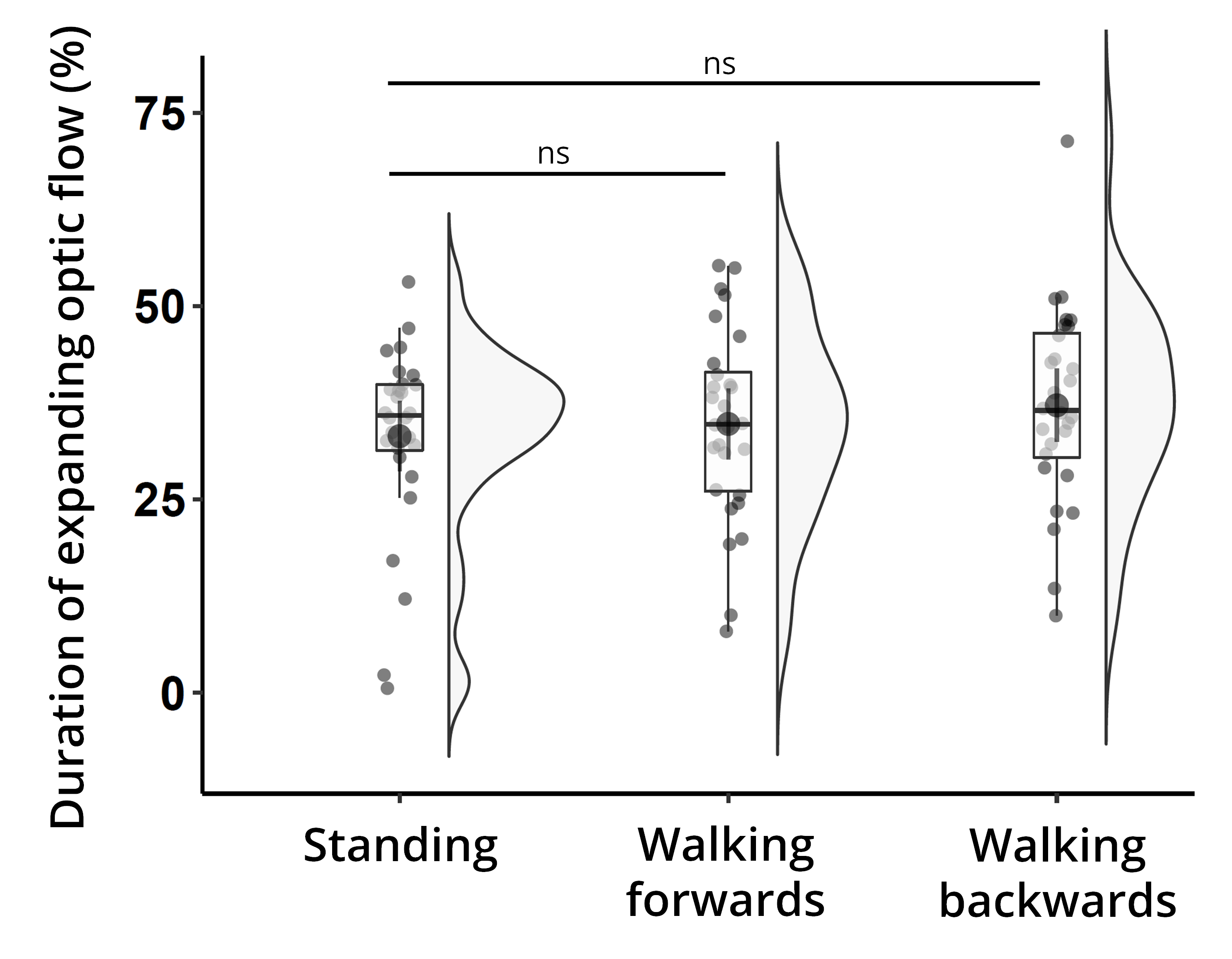


**S6 Fig. Perceptual awareness of expanding optic flow while standing and walking in different directions.** No significant differences were found between training (standing) blocks and visually identical blocks with either forward or backward self-motion.


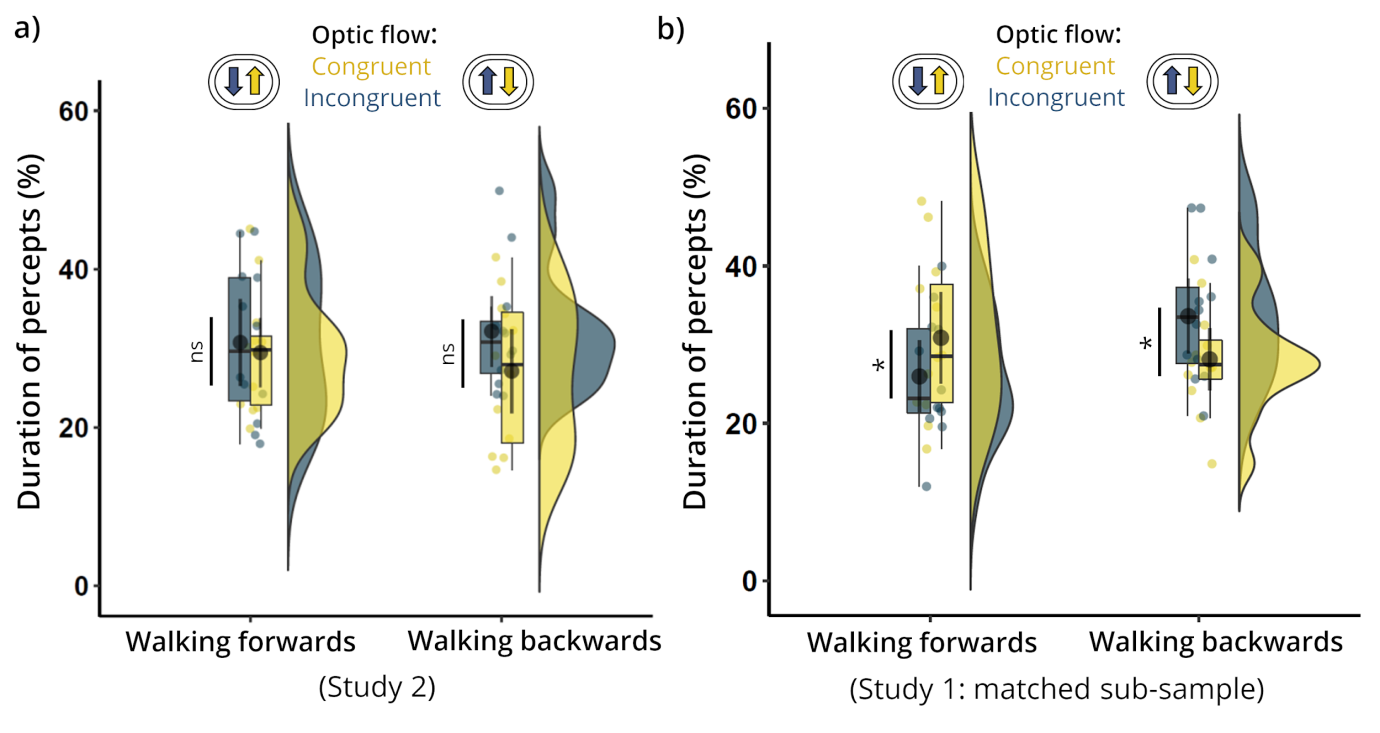


**S7 Fig. Effects of locomotion on perceptual awareness of optic flow patterns for both studies.** (**a**) Study 2: when contracting optic flow (congruent with backward locomotion) was faster than expanding flow (congruent with forward movement), no significant differences in cumulative durations of percepts were found in either walking condition (p values > 0.283). (**b**) Study 1 (sub-sample matched with respect to the distribution of mixed percepts in study 2): when expanding optic flow was faster than contracting flow, it dominated visual awareness independently of walking direction (p values < 0.022). * p < 0.05; ns = non-significant.
