# Supplementary material for: Forward optic flow is prioritised in visual awareness independently of walking direction
Source: PLoS One. 2021 May 4;16(5):e0250905. doi: 10.1371/journal.pone.0250905 (PMC8096117; doi:10.1371/journal.pone.0250905)
Supplement: Attachment — Submitted filename: Response to Reviewers.docx [file pone.0250905.s011.docx]

**Response to the Reviewers**

We thank the editor and the reviewers for their thoughtful comments and constructive suggestions. We have addressed all of them and believe that they have substantially improved the manuscript. In the following, the reviewers’ comments are written in dark gray *italics* and our responses in plain text. Changes made in the revised manuscript are highlighted in color.

**Reviewer 1 – comments and responses**

*This is a follow-up on a very intriguing finding by Paris et al (2017), who had found, unexpectedly, that physical walking direction (backwards or forwards) does not bias competing patterns of optic flow.

The present study reproduces and extends upon this finding. With respect to Paris et al, authors have used a larger number of subjects. They also aimed to reduce the perceptual-motor mismatch between the flow stimulus and walking. For this they used treadmill walking rather than circular walking, and a structured flow pattern rather than a cloud of points. The results fully confirm the intriguing unexpected finding of Paris et al.*

*Over all, the paper is very well written, and provides a good overview of the literature. The methods are well described and the statistics are fine. Probably due to a misunderstanding of the instruction a large number of subjects had to be excluded but the supplementary results show that excluding these subjects this does not affect the conclusion.*

We thank the Reviewer for this synthetic summary and for highlighting the strengths of the manuscript.

*There is one major issue, i.e., that the Discussion is too superficial and misses a number of important points. Although the authors do give an interesting but rather complicated and far-fetched explanation for the lack of an effect, they fail to discuss earlier explanations (e.g. by ref 31, Paris et 2017), as well as simpler explanations, as explained in detail below. Moreover, a conclusion is presented that is not supported by the data (see below).*

*Overall, the data are important and interesting, but a simple explanations should be favoured over complicated ones.*

We have carefully considered all the Reviewer’s suggestions and made substantial changes to the discussion (please see our point-by-point replies to particular comments below). Most importantly, we have included a new paragraph describing suggested methodological considerations that could provide a simpler explanation of the observed results (starting line: 586; also pasted under the section of relevant comments). This part now precedes our initial interpretations, which we believe helps balance the emphasis on different ideas, with the less speculative ones being discussed first. In general, we agree that too much focus was put on a Cancellation theory-based explanation of the results, which, unjustifiably, resonated also in the conclusion. The part covering this interpretation has been rewritten in a much shorter form, which in our view is also more adequately nuanced and clearer now. We have now made sure not to present the Cancellation theory as the favored interpretation of our data, but rather as a speculative take on emerging findings in the domain of interest (starting line: 604; also included below). Finally, the conclusion – both in the abstract and the discussion – has been revised to enunciate more directly what our findings suggest, instead of speculating on how they might be linked to different theoretical accounts.

Revised conclusion (abstract; line 25):

“(…) These predictions were not confirmed: optic flow consistent with forward locomotion was prioritised in visual awareness independently of walking direction and proprioceptive abilities. Our findings suggest the limited role of kinaesthetic-proprioceptive information in disambiguating visually perceived direction of self-motion and indicate that vision might be tuned to the (expanding) optic flow patterns prevalent in everyday life.”

Revised conclusion (discussion; line 584):

“In conclusion, under the conditions used in this study, we did not find evidence for increased perceptual awareness of optic flows congruent with direction of walking. Our findings suggest that kinaesthetic-proprioceptive processing might play a negligible role in clarifying the visually perceived direction of self-motion. Instead, the observed results indicate that visual processing might be tuned to visual flows consistent with our life-long experience of forward locomotion.”

***MAJOR***

*Discussion, second paragraph (line 515-535)*

*Line 515-516: This sentence is not clear. What is meant with “These results […]”? Apart from ref 31, the cited studies are not about locomotion, but rather on hand- (ref 23, 26) and eye movement (ref 27). The authors seem to suggest that action does not influence bistable percepts, but this cannot be so because in (31) it does make a difference if the subject sits or walks, and in (ref 23), there was an influence on perceived rotation direction. Moreover, the authors should refrain from claims about “most previous reports”, while citing just four studies.*

We thank the Reviewer for pointing out the imprecisions in this sentence. Even though it was meant to be a general opening of the paragraph regarding action effects (with the closer specification of referred findings in the later part), we fully agree that it was indeed misleading. We decided to remove this sentence and to revise later discussion of prior findings with regard to the Reviewer’s points (please see the revised paragraph under the last comment referring to this section).

In general, we made sure to clearly denote studies employing locomotion and manual movements (which, notably, were also present in the study by Veto et al., 2018; ref. 26; noted as 27 in the comment above). Second, we stressed that we limit the discussion to the “classical” spatial congruence effects (which were the most relevant, given our interests in this study). For this reason, we have refrained from discussing the overall effect of self-motion – in any direction – on the mean duration of exclusive percepts (alternation rate) at that point (Paris et al., 2017; ref. 31; as we considered this to not be directly related to our hypothesis). Further, we explicitly restricted the discussion to studies using interocular suppression paradigms (i.e., binocular rivalry, onset rivalry, and continuous flash suppression; although we have added an annotation indicating that bistable motion was also used in a study by Dogge et al., ref. 23). Notably, the action-related congruence effects observed in paradigms not involving perceptual suppression were already referred to in the introduction (line: 53).

*Same paragraph, Line 528-530: “with one exception” is misleading. First: science is not a democratic business, and, even then, a majority of three to one can hardly be regarded as a convincing argument.*

We thank the Reviewer for this remark. The relevant fragment has been adjusted accordingly based on this suggestion (line: 563).

*-Same sentence: “local”: this seems to be an arbitrary and ad-hoc category of movements.*

We agree with this point. The term “local” has been removed from the paper.

*Same paragraph, Line 533-535: states a conclusion:*

*“Therefore, the cumulative evidence suggests that – at least in interocular suppression-based paradigms – bodily actions do not favour an intermodally coherent interpretation of visual signals, or such effects are much less robust than in case of exteroceptive influences.”*

*This conclusion is not supported by the cited data. First: ref 25 cites an opposite finding (the authors seem to think that the finding is somehow invalidated by being “not recent”). Second, as note above, refs 23 and 31 also present examples of action that does influence the percept. Third, the authors cannot claim in a general sense that “intermodally coherent interpretation” is not favored in even for their own results because they ignore the “mixed percepts” even though these amount amount one fourth of the response time.*

We thank the Reviewer for these clarifying remarks. The discussion of prior literature has been nuanced so to avoid the above-described ambiguities and to precise which observations from these studies are being referred to (see also our response to the first comment in this section). Additionally, the conclusion has been changed accordingly, to (what we believe) could be more safely concluded based on the review of literature on crossmodal influences on binocular rivalry. For the discussion regarding mixed percepts, please see our response to the relevant comment below.

(starting line: 549)

“Our results seem to be at odds with Bayesian accounts of perception according to which the brain uses probabilistic information to optimise inferences about the state of the environment [38, 39, 61]. Prior expectations – in the form of learned associations between multisensory signals – bias the interpretation of unimodal sensory input towards percepts congruent with information available across different senses. Such effects of signal relatedness have been convincingly demonstrated for binocular rivalry in the domain of exteroception [8–16]: for example, simultaneous auditory and tactile influences can sum up to boost the perceptual availability of an intermodally congruent visual image, but cancel each other out in the case of conflicting audio-tactile stimulation [11, 62]. Given that, in everyday life, we are exposed to a certain repertoire of visuomotor associations (e.g., our visual field expands while moving forwards and contracts while moving backwards), one might expect similar congruence effects in the domain of action, namely increased perceptual awareness of stimuli representing the likely consequences of one’s actions. However, the evidence from prior studies that used perceptual-suppression based paradigms is mixed. The null findings in the present study converge with previous research, which did not demonstrate enhanced visual awareness of locomotion-congruent optic flow patterns [31]. For rotatory hand movements, significant effects were found in a study by Maruya et al. [25], while other studies did not reveal increased perceptual access to stimuli rotating in congruence with manual actions [23, 26, 27; but see also 23 (study 2) for effects in a bistable motion paradigm that did not involve interocular suppression]. Whereas some of the discrepancies might be due to methodological differences between these studies [26, p. 8], the cumulative evidence seems to suggest that congruence effects due to bodily actions are either very context-sensitive or much less distinct than in the case of the widely-reported exteroceptive influences on visual awareness [8–16].”

*The authors do present an interesting explanation for their results on the basis of cancellation theory. It should be noted though, that cancellation theory describes a very different case, i.e., it is about sensorimotor consistence. Put in a simple way: as long as the self-stimulation is consistent with the predicted outcome, it does not lead to a prominent perceptual experience. However in the present results there is an inconsistency, and so it is not at all obvious that it can be explained with the cancellation theory.*

*Rather, the authors need to discuss the following aspects:*

*– The thesis/explanation of Paris et al. (ref 31) to explain the phenomenon should be discussed in the light of the present experiments: “Perhaps this reflects a coarser form of action/perception link than we originally envisioned”.*

Paris et al. (2017; ref. 31) put forward an interesting interpretation of the relatively longer mean durations of individual percepts (decreased number of perceptual switches) during both walking conditions compared to the sitting condition. The authors tentatively suggested that this might be due to the (“higher-level”) expectation of observing radial visual motion – without a specified direction – with one’s locomotion (which, they suggest, could be further explored by including conditions with externally-driven self-motion). While this is indeed an interesting idea, we believe that it does not seem to apply to our discussion of why we did not find support for the hypothesised congruence effects (assessed by mean *cumulative* dominance durations of particular optic flows under different walking conditions). Therefore, we do not see how the currently reported results could provide insight into their theoretical question, which would seem to require the use of a very different experimental design.

However, we would like to note that following the other Reviewer's question, we ran additional analyses to check whether there was an indication that a coarser form of action/perception link could underlie the obtained results. More specifically, we examined whether the primary effect of enhanced perceptual awareness of expanding visual flow differed between standing (training) blocks and matched experimental blocks featuring self-motion. We found no evidence for the modulation of cumulative dominance times by mere fact of movement in the available data (starting line: 432).

As for the point regarding Cancellation theory, we fully agree that this account has been developed mainly in the context of self-produced somatosensory sensations (and not bistable perception-based paradigms), but it seems increasingly frequently discussed in the context of the visual processing of expected consequences of one’s actions (e.g., Yon & Press, 2017; Cardoso-Leite et al., 2010). In the new version of this paragraph, we did our best to clarify the rationale behind applying this approach to other (e.g., visual) sensory domains. Also, we briefly indicate its explanatory limitations in the context of various multisensory processes, in which kinesthetic-proprioceptive information clearly plays a significant role. The revised/new paragraph now reads as follows:

(starting line: 604)

“Alternatively, a more overarching speculative interpretation based on Cancellation theories of perception [66–68] could perhaps explain the scarcity of congruence effects in the domain of action influences on visual awareness [23, 26, 27, 31, but see also 25]. According to this approach, the processing of self-generated sensory signals becomes suppressed in order to prioritise more informative (unexpected/uncontrolled) events. This theory is traditionally used to explain why self-administered tickling [66, 69] or putting pressure on one’s own body [70, 71] are perceived as being less intense than when equivalent stimulation is produced externally.  However, it can also be applied to other sensory domains, for example, to interpret the effects of reduced visual sensitivity to expected consequences of one’s actions [72, 73]. In line with this view, the influences of action-based predictions are suppressed as a redundant source of information about the state of the environment (see also [74]). Perhaps, this could explain why motor actions do not yield comparable congruence effects to those of exteroceptive stimulation or bodily signals that did not follow from voluntary movements – such as externally-produced whole-body rotations [18] or passive proprioceptive signalling of one’s limb position [19]. However, this is not to say that, during voluntary movements, the influences of kinaesthetic-proprioceptive signals are generally suppressed in multisensory integration processes. For example, there is growing evidence for the widespread distribution of locomotion-related signals in the primary visual cortex (e.g., [75]; for reviews see [76–78]) and the modulation of the visually perceived self-motion speed by one’s locomotor activity [79–82]. Notably, the processing of signals evoked by the stimulation of proprioceptive (muscular) nerves has been shown to be actually facilitated during voluntary movements (as opposed to cutaneous inputs which are then suppressed) [83]. More broadly, it might be suggested that while integration of proprioceptive-kinaesthetic and visual cues seems to play a limited role in disambiguating the perception of external (e.g., visual) signals, it primarily serves other (body-oriented and not necessarily perceptual) multimodal processes, such as action control [84, 85], localisation of body parts [86, 87], determination of body ownership [88, 89] and peripersonal space [90, 91].”

*– The legs are little represented in the cortex (much less than hands, mouth, face), and so, there might little perceptual coupling for that reason.*

We thank the Reviewer for this interesting comment. Whereas the legs indeed have relatively less representation in the primary somatosensory cortex than the hands or face, it could be noted that processing of self-motion is not limited to afferent somatosensation. For example, growing evidence from animal studies indicates the widespread presence of locomotion-related signals – originating from a motor-related area of the cortex (A24b/M2; Leinweber et al., 2017) – in the early stages of visual processing (for reviews see: Busse, 2018; Chaplin & Margrie, 2020; Parker et al., 2020; for most comparable results in humans see: Cao et al., 2020; Cao & Händel, 2019), which might be of even greater importance for the biological plausibility of locomotion effects on visual awareness. As we based our hypothesis on prior evidence of crossmodal effects (in the framework of Bayesian models of multisensory integration) and also did not collect any neurophysiological data, we hesitate to go into a detailed (and necessarily speculative) review of different neurobiological underpinnings speaking either in favour of or against possible walking effects on the rivalry.

*– The asymmetry of forward-backward responses and the unusual occurrence of backward visual flow and backward propulsion (except rowers and reverse runners) might mean that the “backward” percept in fact was not the counterpart to the “forward” percept, but rather, that the “backward” responses were among the “mixed” percepts: the “backward” responses might thus rather represent a “contracting” percept, and the participants simply pressed the button for they would otherwise hardly ever press it (this might be consistent with Fig 3b lower panel, where the mixed and backward percept seem to alternate).*

We believe the Reviewer’s comment could be split in two interrelated points: (1) the unusually rare “backward” (contracting) percept cannot easily be considered a natural counterpart of the “forward” (expanding) visual flow; and (2) perhaps the participants might have been biased to either categorize the “backward” percept as mixed or even perceive it as less exclusive than the “forward” one when making the respective button presses. We thank the Reviewer for this interesting comment – if we have misinterpreted it, further clarification would be much appreciated.

As for the first part, we acknowledge the imbalance in lifetime exposure to different visual flows (which indeed might underlie the observed preference for expanding visual flow; line: 654), but, at the same time, we were mainly interested in creating a typical design based on presenting percepts that are either congruent or incongruent with non-visual factors of interest. Even if the backward percept did not function as a counterpart, we should still have observed increased dominance of forward optic flow while walking forwards as compared to walking backwards (if crossmodal congruency effects were there to be found). Yet, as we found no evidence of the influence of walking direction, it is hard to speculate based on the given data (and also on the study by Paris et al., 2017) that any optic flow was perceived/interpreted as different in terms other than the direction of motion.

Nevertheless, we did our best to further address this question by assessing whether response patterns in the case of forward percepts differed from the backward ones, which would potentially support the view they were not perceived as counterparts (e.g., were not reaching similar levels of exclusive visibility). For this purpose, we analyzed the data on mean duration of individual percepts as well as the number of particular presses (for forward/backward optic flows). In line with the main results, we found that, when participants were walking forwards, the mean duration of forward percept (M = 2.51, SD = 1.02 s) was longer than mean duration of the backward one (M = 2.13, SD = 0.84 s; p < 0.001; Bonferroni corrected comparisons). The same was observed for the walking backwards condition (forward percept: M = 2.70, SD = 1.31 s; backward percept: M = 2.40, SD = 1.20 s; p = 0.004). Importantly, we did not observe significant differences between individual numbers of forward and backward responses per block (independently of their duration) in both walking conditions (forward locomotion – forward percept: M = 18.8, SD = 6.33; backward percept: M = 18.7, SD = 6.34; p = 1.000; backward locomotion – forward percept: 18.7, SD = 5.95; backward percept: M = 17.80, SD = 8.35; p = 1.000; Bonferroni corrected comparisons).

We believe that these results preclude the possibility of two unusual response pattern scenarios (presented in simplified form in Fig 1a,b) which might indicate that perception/response criteria for “backward” optic flow were markedly different than for the forward percept – importantly both of them would lead to overall effects of longer proportional dominance (%) of forward optic flow.

Fig. 1. Possible course of binocular rivalry in two scenarios of unusual response patterns.

First, it could be that prolonged periods of mixed percepts appeared interchangeably with (“instead of”) prolonged periods of backward percepts. Such a scenario (a) would be supported by the observation of a lower number of “backward responses” as compared with “forward” ones and no evidence for longer duration of individual “forward” response. Second, it could be that “forward” responses were associated with exclusive dominance of expanding visual flow, and participants continuously alternated between “mixed” and “backward” responses in the remaining periods (scenario b: e.g., due to relatively worse visibility of the latter). Such a course of rivalry could be supported by the conjoint observation of longer durations for individual “forward” responses (as observed) and a relatively greater number of brief “backward” presses. Instead, the observed results seem to indicate the presence of a stereotypical course of the rivalry with prolonged durations of one of the stimuli (with comparable variances of duration case for both types of percepts). Nevertheless, we admit that a “hard” question about what observers actually perceive while pressing particular buttons is unlikely to be resolved based on behavioral data. Yet, in our view, when understood in this sense, it seems to touch on a broader issue applicable also to other binocular rivalry studies (which do not involve no-report paradigms or measurement of the neurophysiological correlates of perception).

*– Possibly the walking speed was too slow: especially at slow speeds, the central field will hardly contribute to a percept of self motion so that the lack of peripheral view is problematic in the present study.*

We thank the Reviewer for this comment. Even though locomotion on a treadmill is felt as being much faster (21–62%) than equivalent locomotion on the ground (Dal et al., 2010; Kong et al., 2012; Kong et al., 2009), we nevertheless acknowledge that it could have been unnaturally slow, so we have identified this as one of the methodological limitations in a newly introduced paragraph (line: 587, included also later under the last design-related comment).

Notably, in this study, a relatively wide peripheral view was actually used (110° for both horizontal and vertical axes – line 159 in the methods) to preserve the feelings of speed – the employment of even wider displays would probably be unfavourable due to the increasing tendency for piecemeal rivalry on visual peripheries. Yet, given the restriction of the visual field in head-mounted displays as compared to normal vision, the presented optic flows needed to be faster to appear subjectively accurate (Banton et al., 2005; Caramenti et al., 2018; Caramenti et al., 2019; Durgin et al. 2005; Powell et al., 2011) – which we took into account by performing an additional pre-study. The subjective matching of visual flow to walking speed ascertained that visually-perceived self motion was clearly conveyed by the final stimuli.

*– Since the hand held the side-rim of the treadmill, the subjects had a very strong perceptual reference of the stationary world, which might have additionally weakened the percept of self-motion. This would probably weaken a possible influence of walking direction.*

We thank the Reviewer for this important remark. We have integrated this point into a new paragraph discussing limitations of the current design that could have resulted in the lack of congruence effects in the present study (line: 589).

*– Virtually all people are subject to regular driving experience, and in this case, they receive a strong expanding flow in the central visual field without active movements. This might cause some extent of decoupling of locomotion and optical flow in the central field.*

We thank the Reviewer for this interesting comment. Indeed, it could be the case that lifetime experience of observing visual expansion even without active self-motion (e.g., in a car) could have relaxed overall visuomotor associations pertaining to optic flow perception. Nevertheless, this would imply that regular driving experience would be sufficiently influential to diminish an otherwise-likely-to-be-observed congruency effect. In our view, this would make an interesting example of strong generalization of a less frequent and context-specific association (observing visual flow despite being seated) to a much more common and markedly different context of active walking (virtually always associated with highly-predictable optic flow changes). That being said, while the extent to which the experiences of functioning in the modern world are reshaping expectations of crossmodal/visuomotor consistencies is an intriguing question, it seems to be beyond the scope of this study.

*– The stimulus might still not be realistic enough / correspond well enough: It had no rhythmic lateral sway nor anteroposterior acceleration in phase with walking. (But see arguments against this by Paris et al.).*

In the new paragraph discussing methodological considerations, we acknowledge that the visual motion did not accelerate according to the gait cycle (line: 591), which indeed is an important limitation of the used treadmill-based setup. Undoubtedly, natural body movements (e.g., medio-lateral trunk sway) were also partially constricted due to the requirement of pressing the buttons. Notably though, the position of perspective in the virtual scene was not fixed on horizontal and vertical axes, but rather in both eyes it shifted according to rhythmic head movements while walking (this fact was highlighted also in the revised discussion: line 591). We considered it necessary to evoke feelings of being in a virtual scene and perception of visual flows as likely results of one’s bodily movements. To assess whether head movements indeed showed the typically-observed pattern during self-motion, we analysed the data on continuous changes in headset position (i.e., rhythmic yaw [left-right] and pitch [up-down] rotations). We found distinctive and analogous patterns of head motion in both walking conditions (please see Fig. 2 for sample data on yaw head rotation).

Fig. 2. The left panel presents the raw data indicating head rotation along the horizontal axis (black points) with the fitted (blue) smooth. The left right panel shows the same interval with estimated turning points of each head rotation (colored peaks).

*– It could be that the displayed movement did not convey enough self-motion, i.e. was still too much expansion-contraction like: It presented no visual periphery, and did not follow the rhythmic sway of walking.*

We believe that all aspects of this remark have been addressed in the responses above. Overall, we thank the Reviewer for all of the methodological remarks, which made us acknowledge that simpler explanations should also be discussed, thus providing a more just and comprehensive understanding of the observed results. The new paragraph describing simpler explanations of the observed results reads as follows:

(starting line: 586)

“Speculatively, methodological factors could also have contributed to the lack of bodily movement effects on visual experience in the present study. Even though locomotion on a treadmill is felt as being much faster than on the ground [46, 64, 65], it still might have been that the action effects were hindered by the use of unnaturally slow walking speed. The fact that the participants’ hands held the side-rim of the treadmill could have additionally contributed to weakened overall feelings of locomotion. Despite the visualisations of self-motion being presented at subjectively realistic speed (including mid-peripheral vision) and coupled to rhythmic head movements while walking, the visual motion did not accelerate/decelerate according to the concurrent phase of the gait cycle. This constitutes another palpable difference between the current setup and multilevel cohesion of visuomotor signals under natural walking conditions. Perhaps the combination of such lesser-scale inconsistencies could have prevented the generalisation of learned (broader-level) sensorimotor associations to distinctively unfamiliar sensory conditions. These limitations could mostly be overcome with the use of a wireless VR headset in a space allowing for long periods of unidirectional walking. On the flip side, as thoroughly discussed by Paris et al. [31, p. 1192], multimodal effects on binocular rivalry have been demonstrated in studies utilising less realistic conditions, which seems to suggest that the issue of detectability of such effects cannot easily be reduced to the ecological validity of crossmodal (e.g., visuomotor) correspondences."

***MINOR***

*Title: it would be good if “binocular rivalry” occurred in the title.*

If this is a minor suggestion, we would respectfully prefer to keep the original title. In its defense, we would like to note that: a) binocular rivalry is described already in the first sentence of the abstract; b) “visual awareness” seems to be often used in the literature to signal the involvement of interocular suppression paradigms (e.g., in titles where they are not explicitly mentioned); and c) in our view, further extension (e.g., adding “during binocular rivalry”) would make this particular title less reader-friendly/too overloaded.

*Line 89:*

*It is claimed that the structured environment is an improvement, but Paris et al (2017) did test with structured environments: “Moreover, in a supplemental experiment (Supplement 1), we replicated the main results using, instead of dots, textured wall and ceiling surfaces to present dissimilar perspective-defined optic flow patterns to the two eyes.”*

While we truly appreciate the solidity and scope of work done by Paris et al. (2017) assessing walking effects on binocular rivalry, we nevertheless saw room for improvement in developing more realistic and detailed visual stimulation. We actually started the pilot phase with designs similar to the [textured one](https://static-content.springer.com/esm/art%3A10.3758%2Fs13414-017-1299-4/MediaObjects/13414_2017_1299_MOESM1_ESM.pdf) used by Paris at al., but, in the case of our observations, the flat surfaces conveyed much weaker feelings of self-motion. The patterns drawn on these surfaces were also spatially-mismatched between the eyes when flowing forwards/backwards, preventing their full perceptual suppression. Compared with these initial designs, the final one had the benefit of evoking a more realistic experience of self-motion through actual space.

Nevertheless, as the previous version could have mistakenly suggested that only dot motion stimuli were used in the study by Paris et al. (2017), we decided to add a concise clarification in a fragment discussing the differences between the stimuli used in both studies. This fragment now reads:

(line: 89):

“First, instead of presenting shifting dots, we used a more realistic three-dimensional environment to boost general feelings of presence and locomotion through space (although it should be noted that in a supplementary experiment by Paris et al., stimuli presenting a hallway with patterned flat surfaces were used [31, Fig. S1])”.

*Dissociation/decoupling due to lack of gait-associated lateral movements and anteroposterior accelerations in the display?*

Please see our earlier response to a closely related comment (starting with *“The stimulus might”*).

**Reviewer 2 – comments and responses**

*This study by Motyka et al investigates the effect of walking direction on the perception of optic flow direction during binocular rivalry and its modulation by proprioceptive precision. Building up on the literature showing that signals from different sensory modalities can bias visual perception during binocular rivalry in favour of the visual stimulus congruent with the cross-modal one, the authors used a sophisticated and well controlled virtual reality setup in which binocular rivalry was generated between opposite optic flow directions (consistent with forward and backward walking direction) while participants walked on a treadmill either forward or backward. The main result of the study (in line with previous evidence) is that walking direction does not impact visual rivalrous perception, and that forward optic flow consistently dominates binocular rivalry independently of the walking direction. The authors interpret this null result in the framework of the cancellation theory.*

*The study is well conducted and controlled, the authors made a technical tour de force to design the experimental setup using VR and controlling visual stimulation velocity. The paper is well written and the relevant literature is well discussed.*

We thank the Reviewer for the thoughtful review and positive feedback on our work.

*My main concern about the study is the lack of a baseline condition in which binocular rivalry between the two optic flow directions is measured while participants simply stand (I understand that they used this condition during the training, but they only recorded two 45-sec blocks per participant). I believe that measuring the intrinsic bias for the forward flow preference could be helpful to normalize for each participant the data recorded while walking to a baseline, visual-only, condition. The effect of walking direction (probably because of the cancellation theory) might be very small and concealed by the intrinsic visual bias, but without a baseline condition, the bias migh conceal the effect. Walking backwards is a highly unnatural condition, it is therefore possibe that the effect is present only for the forward direction, but it is impossible with the current data to disentangle the visual perceptual bias from the possible cross-modal effect.*

*An efficient way of testing this possibility and maximizing the effectiveness of cross-modal stimulation could be that of interleaving within an experimental block relatively short (eg. 20-30 sec) periods of walking and rest.*

*I understand that it might be very challenging in this period to perform an additional experiment, but I really think that it might be crucial to interpret the results correctly.*

We thank the Reviewer for raising this important question and suggesting the means to empirically examine it. We agree that inclusion of a standing (baseline) condition controlling for the role of self-motion *per se* (independently of its direction) would allow quantification of the baseline preference for forward/expanding optic flow and disentanglement of existing visual bias from possible action effects. However, we are not convinced that any specific differences between standing and walking conditions would substantially change the overall interpretation of the currently observed results. In our view, the hypothesised differences should still be observed in the current design if congruence effects were at work; that is, perceptual dominance of expanding optic flow would be found to be greater while walking forwards than while walking backwards (even despite the presence of overall visual bias). Even so, we are convinced that inclusion of a standing (baseline) condition would certainly help to clarify whether self-motion played any role in shaping dominance durations.

As pointed out by the Reviewer, one possible outcome of newly run comparisons could be the observation of decreased preference for forward optic flow in a baseline (standing) condition relative to walking conditions. We believe that such a finding might have two plausible alternative explanations. (1) This could be interpreted as evidence that the congruence *effect is present only for the forward direction*. Yet, in our, view, such an interpretation could be defended either by making the additional assumption that walking backwards is, in principle, incommensurable with the two other conditions (which after all could be true but seems a bit ad-hoc/arbitrary) or by providing a specific explanation of why the observed preference for expanding flow was also observed while walking backwards (obviously different from the one based on congruency effect). (2) Perhaps such a finding could also be interpreted as an indication of a coarser form of self-motion effect; that is, relatively enhanced perceptual access to visual flow consistent with life-long experience of locomotion (i.e., expanding) independently from the exact direction of self-motion.

Unfortunately, due to the current pandemic situation in Poland (i.e., no prospect of running VR-based studies in a foreseeable future), we could not address the Reviewer’s question by collecting additional data. Yet, to at least partially inform whether the observed results were only due to the intrinsic visual bias or rather to a combination of the bias and the influences of self-motion, we did our best to make use of the data available from the standing (training) blocks. Given that there were only two of these (shortened) blocks per participant (featuring random-set of visual factors: line 243 in the method), we hesitated to perform normalization of the (supposedly more reliable) data from the experimental (fully counterbalanced) blocks, using data obtained during the training phase. Instead, following the main question of interest, we aimed to compare dominance durations of expanding optic flow between two training blocks and the matched of experimental blocks with equivalent combinations of visual factors. To further increase their comparability, the duration of these paired blocks was shortened to the length of the training blocks. As a result, the obtained data included two sets of three visually identical 45-second blocks per participant (with each set consisting of standing, walking forwards, and walking backwards conditions). To assess whether the predominance of expanding visual flow differed significantly between the standing and either of the walking conditions, focused comparisons along with corresponding Bayes factor analyses were used.

This new analysis has been included as the last subsection to the results of the main study and has also been pasted below.

(starting line: 432) **“Comparison of expanding optic flow dominance while standing and walking forwards/backwards – supplementary analysis**

The fact that, instead of the hypothesised congruence effects, we observed perceptual prioritisation of expanding visual flow in both walking conditions might raise the question of whether these results were merely due to the intrinsic visual bias or rather to a combination of the bias and the influences of self-motion (we thank an anonymous reviewer for bringing this point to our attention). To at least partially address this question with the available data, we compared dominance durations of expanding optic flow between two training blocks (in which participants stood still) and experimental blocks with equivalent combinations of visual factors. As each combination occurred once in both walking conditions, particular training blocks could always be matched with their  forward and backward self-motion counterparts. Additionally, the duration of these paired blocks was shortened to the length of the training blocks (so as to level out the relative significance of the initial preponderance of mixed percepts; cf. Fig 3a).  Exploratory pairwise comparisons were run to examine differences in dominance durations of expanding optic flow between a) standing and walking forwards, and b) standing and walking backwards (n = 28, due to missing training data from one subject).

The dominance of expanding optic flow while standing (M = 33.2%, SD = 12.2%) was not significantly different from its dominance when walking forwards (M = 34.8%, SD = 12.4%, t(27) = −0.55, p = 0.587) or backwards (M = 37.2%, SD = 12.7%, t(27) = −1.46, p = 0.157; S6 Fig). Furthermore, to assess whether these non-significant results reflected genuine null effects rather than insufficient statistical power, the corresponding Bayes factor analyses were performed (Dienes, 2014; Morey & Rouder, 2018 – using the default Jeffreys–Zellner–Siow prior, as we lacked justification for informed prior specification).  As for the comparison between standing and walking forwards, there was substantial evidence for the null hypothesis (BF_10_ = 0.23). However, in the case of comparison between standing and walking backwards, we found only anecdotal support for the null effect (BF_10_ = 0.51). These analyses suggest that forward self-motion does not enhance perceptual awareness of expanding optic flow relative to the stationary position. Similar support for the null effect cannot be fully concluded for the case of walking backwards. Nevertheless, altogether, these exploratory results do not yield indications that the primary effects of this study could have been caused by a specific/coarser form of visuo-motor interactions (e.g., increased predominance of a more familiar visual flow with any type of self-motion).”

**“Fig S6. Perceptual awareness of expanding optic flow while standing and walking in different directions.** No significant differences were found between training (standing) blocks and visually identical blocks with either forward or backward self-motion.”

We are aware that this post-hoc (not pre-planned) analysis does not mirror exactly the one that could ideally be run on a full set of equally reliable standing blocks. We agree that the inclusion of additional interleaving visual-only conditions would more directly address the questions discussed above, allowing normalization of responses recorded while walking to individual baselines derived from equally reliable (non-training) and counterbalanced standing blocks. Nevertheless, we do believe that our supplementary analysis addresses (at least in an approximation) seemingly the most pressing question of whether the observed predominance of expanding visual flow was only due to intrinsic bias or partially also due to self-motion influences (e.g., the presence of congruence effect only for walking forwards). Even though the results seem to unequivocally favour a “visual-bias only” interpretation, we note that, given the limitation of available data, this finding should certainly be viewed with caution.

Additionally, after the consideration of the other Reviewer’s comments as well as the overall limitations of the current design, we changed the conclusion (both in the abstract and the discussion) to enunciate more directly what our findings suggest, instead of how they might be linked to different theoretical accounts (lines: 25 & 585; included also below). Moreover, substantial changes have been made to the discussion. In short, we have added a new paragraph describing methodological considerations that could provide simpler explanations for the observed results (starting line: 586; included also above in response to Reviewer 1) and rewrote the paragraph on cancellation-theory. We aimed to present it as a speculative take on emerging findings in the domain of action effects on visual awareness rather than a favoured interpretation of the observed results (starting line: 604; included also above in response to Reviewer 1).

Revised conclusion (abstract; line 25):

“(…) These predictions were not confirmed: optic flow consistent with forward locomotion was prioritised in visual awareness independently of walking direction and proprioceptive abilities. Our findings suggest the limited role of kinaesthetic-proprioceptive information in disambiguating visually perceived direction of self-motion and indicate that vision might be tuned to the (expanding) optic flow patterns prevalent in everyday life.”

Revised conclusion (discussion; line 584):

“In conclusion, under the conditions used in this study, we did not find evidence for increased perceptual awareness of optic flows congruent with direction of walking. Our findings suggest that kinaesthetic-proprioceptive processing might play a negligible role in clarifying the visually perceived direction of self-motion. Instead, the observed results indicate that visual processing might be tuned to visual flows consistent with our life-long experience of forward locomotion.”

**The list of additional minor amendments to the manuscript:**

- “We might speculate that, given the negligible contribution of movement-related (proprioceptive-kinesthetic) information to disambiguating the direction of visual motion, the precision of proprioceptive signals – even if it acts as a weighting factor – ends up being practically inconsequential”. (instead of: “to inferences giving rise to visual contents”) [line: 577]

- “This asymmetry-based interpretation seems more suitable for explaining the effects observed in the present research”. (instead of: “alternative”) [line: 663]

- Added information: “Participants were recruited via social media”. [line: 142]

- Due to addition of a new supplementary figure (S6 Fig), the numbering of subsequent figures has been adjusted accordingly.

- The bibliography has been updated to reflect the inclusion of further references during the revision process.

**References:**

Banton, T., Stefanucci, J., Durgin, F., Fass, A., & Proffitt, D. (2005). The Perception of Walking Speed in a Virtual

Environment. *Presence*, *14*(4), 394–406. <https://doi.org/10.1162/105474605774785262>

Cao, L., Chen, X., & Haendel, B. F. (2020). Overground Walking Decreases Alpha Activity and Entrains Eye Movements

in Humans. *Frontiers in Human Neuroscience*, *14*. https://doi.org/10.3389/fnhum.2020.561755

Cao, L., & Händel, B. (2019). Walking enhances peripheral visual processing in humans. *PLoS Biology*, *17*(10),

e3000511. https://doi.org/10.1371/journal.pbio.3000511

Caramenti, M., Lafortuna, C. L., Mugellini, E., Khaled, O. A., Bresciani, J.-P., & Dubois, A. (2018). Matching optical flow to

motor speed in virtual reality while running on a treadmill. *PloS One*, *13*(4), e0195781.

https://doi.org/10.1371/journal.pone.0195781

Caramenti, M., Pretto, P., Lafortuna, C. L., Bresciani, J.-P., & Dubois, A. (2019). Influence of the Size of the Field of View

on Visual Perception While Running in a Treadmill-Mediated Virtual Environment. *Frontiers in Psychology*,

*10*. https://doi.org/10.3389/fpsyg.2019.02344

Cardoso-Leite, P., Mamassian, P., Schütz-Bosbach, S., & Waszak, F. (2010). A new look at sensory attenuation. Action-

effect anticipation affects sensitivity, not response bias. *Psychological Science*, *21*(12), 1740–1745.

<https://doi.org/10.1177/0956797610389187>

Chaplin, T. A., & Margrie, T. W. (2020). Cortical circuits for integration of self-motion and visual-motion signals.

*Current Opinion in Neurobiology*, *60*, 122–128. <https://doi.org/10.1016/j.conb.2019.11.013>

Dal, U., Erdogan, T., Resitoglu, B., & Beydagi, H. (2010). Determination of preferred walking speed on treadmill may

lead to high oxygen cost on treadmill walking. *Gait & Posture*, *31*(3), 366–369.

<https://doi.org/10.1016/j.gaitpost.2010.01.006>

Dogge, M., Gayet, S., Custers, R., & Aarts, H. (2018). The influence of action-effect anticipation on bistable perception:

Differences between onset rivalry and ambiguous motion. *Neuroscience of Consciousness*, *2018*(1).

<https://doi.org/10.1093/nc/niy004>.

Durgin, F. H., Fox, L. F., Schaffer, E., & Whitaker, R. (2005). The perception of linear self-motion. *Human Vision and*

*Electronic Imaging X*, *5666*, 503–514. <https://doi.org/10.1117/12.610864>

Leinweber, M., Ward, D. R., Sobczak, J. M., Attinger, A., & Keller, G. B. (2017). A Sensorimotor Circuit in Mouse Cortex

for Visual Flow Predictions. *Neuron*, *95*(6), 1420–1432.e5. <https://doi.org/10.1016/j.neuron.2017.08.036>

Kong, P. W., Koh, T. M. C., Tan, W. C. R., & Wang, Y. S. (2012). Unmatched perception of speed when running

overground and on a treadmill. *Gait & Posture*, *36*(1), 46–48. <https://doi.org/10.1016/j.gaitpost.2012.01.001>

Kong, P. W., Candelaria, N. G., & Tomaka, J. (2009). Perception of self-selected running speed is influenced by the

treadmill but not footwear. *Sports Biomechanics*, *8*(1), 52–59. <https://doi.org/10.1080/14763140802629990>

Maruya, K., Yang, E., & Blake, R. (2007). Voluntary Action Influences Visual Competition. *Psychological  Science*,

18(12), 1090–1098. <https://doi.org/10.1111/j.1467-9280.2007.02030.x>

Paris, R., Bodenheimer, B., & Blake, R. (2017). Does direction of walking impact binocular rivalry between competing

patterns of optic flow? *Attention, Perception & Psychophysics*, *79*(4), 1182–1194.

<https://doi.org/10.3758/s13414-017-1299-4>

Powell, W., Stevens, B., Hand, S., & Simmonds, M. J. (2011). *Blurring the boundaries: The perception of visual gain in*

*treadmill-mediated virtual environments*. 3rd IEEE VR 2011 Workshop on Perceptual Illusions in Virtual Environments: https://researchportal.port.ac.uk/portal/files/101407/Powell_W_PIVE_2011_final.pdf

Veto, P., Schütz, I., & Einhäuser, W. (2018). Continuous flash suppression: Manual action affects eye movements but

not the reported percept. *Journal of Vision*, *18*(3), 8. <https://doi.org/10.1167/18.3.8>

Yon, D., & Press, C. (2017). Predicted Action Consequences Are Perceptually Facilitated Before Cancellation. *Journal of*

*Experimental Psychology. Human Perception and Performance*. <https://doi.org/10.1037/xhp0000385>
